# Nonlinear Dynamic Analysis of Axially Moving Laminated Shape Memory Alloy Beam with 1:3 Internal Resonance

**DOI:** 10.3390/ma14144022

**Published:** 2021-07-18

**Authors:** Ying Hao, Ming Gao, Yuda Hu, Yuehua Li

**Affiliations:** 1 Department of Engineering Mechanics, College of Civil Engineering & Mechanics, Yanshan University, Qinhuangdao 066004, China; hy0505@ysu.edu.cn (Y.H.); huyuda03@163.com (Y.H.); 2 Department of Mechanical Manufacturing, College of Mechanical and Electronical Engineering, Shandong Agriculture University, Taian 271018, China; 3 Laboratory of Test and Testing Technology, Tianjin Key Laboratory of Microgravity and Hypogravity Environment Simulation Technology, Tianjin 300384, China; yuehua_li88@163.com

**Keywords:** shape memory alloy, axially moving laminated beam, 1:3 internal resonance, nonlinear dynamic

## Abstract

The remarkable properties of shape memory alloys (SMA) are attracting significant technological interest in many fields of science and engineering. In this paper, a nonlinear dynamic analytical model is developed for a laminated beam with a shape memory alloy layer. The model is derived based on Falk’s polynomial model for SMAs combined with Timoshenko beam theory. In addition, axial velocity, axial pressure, temperature, and complex boundary conditions are also parameters that have been taken into account in the creation of the SMA dynamical equation. The nonlinear vibration characteristics of SMA laminated beams under 1:3 internal resonance are studied. The multi-scale method is used to solve the discretized modal equation system, the characteristic equation of vibration modes coupled to each other in the case of internal resonance, as well as the time-history and phase diagrams of the common resonance amplitude in the system are obtained. The effects of axial velocity and initial conditions on the nonlinear internal resonance characteristics of the system were also studied.

## 1. Introduction

Shape memory alloy (SMA) is widely used in machinery, electronics, aerospace, civil engineering, energy, and medicine. With special shape memory and pseudo-elasticity, it is more sensitive to stress and temperature changes and more deformable and resilient than its ordinary metal counterparts. In practical applications, SMA particles, wires, or strips are often inserted into other matrix materials, or an SMA membrane is applied over the surface of a beam or plate matrices to form an SMA composite structure [1].

SMA as the surface and linear-elastic materials as the interlayer make up laminated structures, of which laminated beams are a common form. Machado [2] and Savi et al. [3] investigated the dynamic characteristics and chaotic behavior of coupled SMA oscillators through numerical approaches. Ren et al. [4,5,6] carried out a series of work on SMA composite beams which analyzed the influence of fiber laying angle and the content percentage of SMA on the equivalent damping ratio of the beam, and the vibration frequency response characteristics of the beam structure. Collet et al. [7] considered a symmetry assumption for SMA under tensile, compressive, and temperature loads, and examined the dynamic behavior of the material by applying moving external loads to SMA beams. Odeny D. et al. [8] studied the nonlinearity of the aeroelastic behavior of reinforced SMA hybrid composite (SMAHC) cylindrical plate on a microscopic mechanics model of carbon fiber–SMAHC laminated plate using a layered Rayleigh–Ritz procedure. Akhavan-Rad B. et al. [9] applied the high-order sandwich panel theory to the vibration of SMA wire-embedded laminated plate and provided an analytical solution to the nonlinear control equation for simply-supported sandwich panels under uniform and sinusoidal loading. Reza Razavilar et al. [10] developed a semi-analytical method to examine the free and forced vibration of SMA beams, modeled the control dynamic equation for SMA beams coupled with deformation strain, and analyzed the dynamic characteristics of these beams using phase trajectory.

A large number of constitutive models, including the piecewise-linear model, Brinson’s polynomial constitutive model, and Falk’s model, have been developed to describe the shape memory and pseudo-elasticity of SMA. Lu P et al. [11] proposed a relatively common constitutive SMA–fiber constitutive relation and built a bending theory model of SMA fiber-reinforced composite laminated beams without considering the stress relation between the SMA layer and the matrix layer. Wu et al. [12] developed a singularity theory for multi-piece description of bifurcations on a Tanaka–Liang–Brinson model by describing the delayed constitutive relation of SMA with a flag-shaped piecewise linear function and investigated the nonlinear vibration of SMA laminated beams by building a dynamic model on the basis of their piecewise linear constitutive relation. Brinson’s constitutive model is widely used because of its simplicity and effectiveness. Fu [13] addressed the free vibration of SMA fiber-embedded composite laminated beams using Brinson’s polynomial constitutive model to consider their stress–strain relation. Asadi [14,15] built a control equation for laminated beams with SMA fibers that considered the effects of thermal and aerodynamic loads using Brinson’s polynomial constitutive model; they investigated the thermodynamic behavior of SMA laminated beams.

These investigations have found many interesting and complex dynamics of the laminated beam, in the meantime, nonlinear dynamics with an internal resonance have also been promoted. Zhang et al. [16] examined the 1/3rd subharmonic resonance and 3rd super harmonic resonance of simply-supported SMA laminated beams. Wang et al. [17] established the nonlinear vibration equation of an axially moving conductive beam in the magnetic field through the Hamiltonian principle, then analyzed the nonlinear vibration characteristics of the free vibrating beam under 1:3 internal resonances. Zhang et al. [18] studied the parametric vibration responses in supercritical fluid-conveying pipes in the 3:1 internal resonance condition. Yang et al. [19] considered a composite cantilever piezoelectric plate with one-to-three internal resonance. Zhang et al. [20] investigated the dynamic stability of axially transporting viscoelastic beams with two frequency parametric excitation and 1:3 internal resonance in which the direct method of multiple scales is employed to obtain the solvability conditions in principal parametric resonances. Wang et al. [21] developed a model that proposed an axially moving nano beam with two kinds of scale effects. Based on the root discriminant of the frequency amplitude equation under internal resonance conditions, theoretical analyses are performed to investigate the scale effects of the resonance region and the critical external excitation amplitude. Zhang et al. [22] investigated the instability boundaries of axially accelerating plates with internal resonance. By the method of multiple scales, they established the modified solvability conditions in principal parametric and internal resonances. Hu et al. [23,24] explored the nonlinear dynamics of a super critically moving beam and a traveling viscoelastic beam under the 3:1 internal resonance condition. The direct multi-scale method is used to derive the relationships between the excitation frequency and the response amplitudes. Zhu et al. [25] investigated the nonlinear dynamical behaviors of an axially accelerating viscoelastic sandwich beam subjected to three-to-one internal resonance and parametric excitations resulting from simultaneous velocity and tension fluctuations. Bamadev et al. [26] used the direct method of multiple scales to analyze the joint influence of the combination of parametric resonance and internal resonance with the focus on steady state responses of the axially moving viscoelastic beam. 

An axially moving beam is presently used in a wide range of industrial applications, such as band saws, and power transmission belts. Studies have shown that the beam could oscillate so acutely that the real-life of the device is reduced. However, an internal resonance in the laminated SMA beam has not been reported so far. Hence, the present work aims to investigate the nonlinear dynamics of the axially SMA beam with internal resonances.

An axially moving beam is also a simplified model for many aircraft structures, and its velocity has a significant effect on its vibration characteristics. To determine the internal resonance of axially moving SMA laminated beams, in this paper, we establish a transverse vibration equation for SMA laminated beams under axial loading using the widely accepted continuously smooth polynomial constitutive model. Considering the influence of axial velocity, the multi-scale method is used to solve the nonlinear vibration equation of clamped-hinged SMA laminated beam, and 1:3 internal resonances are analyzed.

## 2. Dynamic Modelling of SMA Laminated Beam in Axial Movement

### 2.1. Polynomial Constitutive Relationship of SMA

Based on the Landau–Devonshire thermodynamics theory, Falk proposed a polynomial free energy equation, then Savi and Braga [27] obtained the coefficients of the free energy function and the constitutive equation is given by:

(1) $$\sigma =a(T-T_{M})\varepsilon -b\varepsilon^{3}+e\varepsilon^{5}$$

where *a* and *b* are positive material constants and $e=\frac{b^{2}}{4a(T_{A}-T_{M})}$. *T_A_* is the temperature above which austenite is stable and *T_M_* is the temperature below which marten site is stable. $a=1\times 10^{3}\;\mathrm{MPa}/K$, $b=40\times 10^{6}\;\mathrm{MPa}$, $T_{A}=313\;K$, and $T_{M}=287\;K$, which were obtained from experiments in Ref. [27], and the stress–strain curve is shown in Figure 4b [27].

The merits of the SMA polynomial constitutive relation lie in its simplicity. Hao et al.’s [28] research also shows that the polynomial model can qualitatively describe the dynamic behaviors of SMA. For a SMA laminated beam with complex structure and complicated stress conditions, it is sometimes difficult to obtain the dynamics equation of the system by using other constitutive models, and the nonlinear dynamics characteristics of the system can be easily obtained and analyzed by using this constitutive model.

### 2.2. Vibration Equation of SMA Laminated Beam

**Figure 1:**
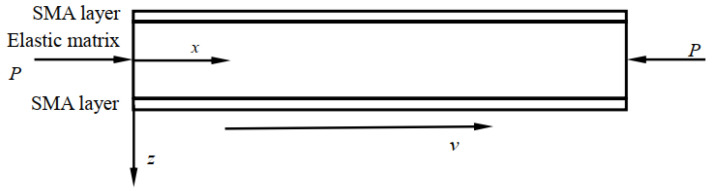
Axially moving SMA laminated beam structural diagram.

Figure 1 provides the structural diagram of a SMA laminated beam. The laminated beam that is subject to axial load P, and the beam’s length is L, width is $b_{1}$; and the height of the matrix beam is H, the thickness of the upper and lower SMA layers is *h*, the axial movement velocity is $\frac{dx}{dt}=v$. Where *oxz* is the stationary coordinate system, with traverse displacement being recorded as w(x,t), the beam traverse movement velocity is $\frac{dw}{dt}=\frac{\partial w}{\partial t}+v\frac{\partial w}{\partial x}$ and the acceleration is $\frac{d^{2}w}{dt^{2}}=\frac{\partial^{2}w}{\partial t^{2}}+2v\frac{\partial^{2}w}{\partial x\partial t}+v^{2}\frac{\partial^{2}w}{\partial x^{2}}$.

Hao et al. [28] gives the force diagram of the micro-body. Considering only the transverse vibration of the beam and omitting the influence of the axial deformation, the transverse vibration equation of the main beam can be obtained.

(2) $$\begin{array}{ll} & -E\frac{b_{1}H^{3}}{12}\frac{\partial^{4}w}{\partial x^{4}}+\frac{H}{2}Eb_{1}H[{\left(\frac{\partial^{2}w}{\partial x^{2}}\right)}^{2}+\frac{\partial w}{\partial x}\frac{\partial^{3}w}{\partial x^{3}}]-b_{1}Hh[a(T-T_{M})\frac{H+h}{2}\frac{\partial^{4}w}{\partial x^{4}}-\frac{3}{8}b{\left(H+h\right)}^{3}{\left(\frac{\partial w}{\partial x}\right)}^{2}\frac{\partial^{4}w}{\partial x^{4}}\\ & -\frac{3}{4}b{\left(H+h\right)}^{3}\frac{\partial^{2}w}{\partial x^{2}}{\left(\frac{\partial^{3}w}{\partial x^{3}}\right)}^{2}+\frac{5}{32}e{\left(H+h\right)}^{5}{\left(\frac{\partial^{2}w}{\partial x^{2}}\right)}^{4}\frac{\partial^{4}w}{\partial x^{4}}+\frac{5}{8}e{\left(H+h\right)}^{5}{\left(\frac{\partial^{2}w}{\partial x^{2}}\right)}^{3}{\left(\frac{\partial^{3}w}{\partial x^{3}}\right)}^{2}]-P\frac{\partial^{2}w}{\partial x^{2}}-\rho b_{1}H\frac{d^{2}w}{dt^{2}}-c\frac{\partial w}{\partial t}=0 \end{array}$$

## 3. Internal Resonance

### 3.1. Differential Equation of Vibration 

**Figure 2:**
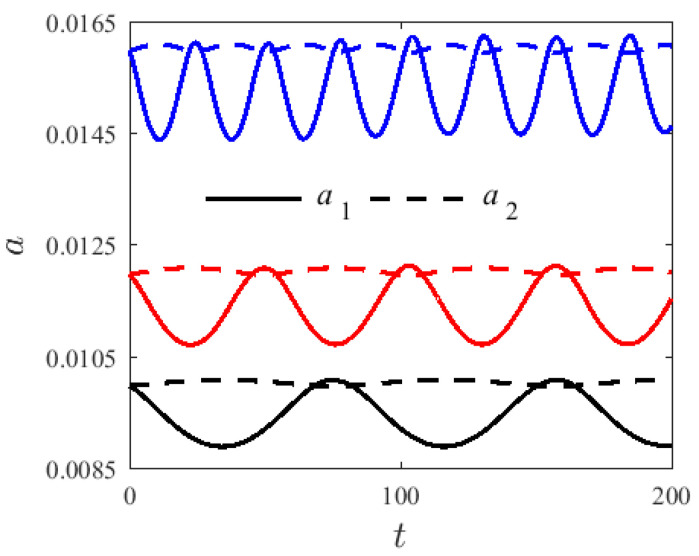
Variation modes for different initial conditions a ($\gamma_{0}=0.2\pi$).

The experimental records from these tests were obtained under constant conditions. The cutting parameters were: the spindle speed of the cutter was 10,400 r/min, the feed rate was 1555 mm/min, the Y depth of cut (radial) was 0.125 mm, and the Z depth of cut (axial) was 0.2 mm. The data were acquired at 50 kHz/channel; the experimental setup is shown in Figure 2.

One end was fixed and the other end simply supported the boundary condition. The expression of the laminated SMA beam is:

(3) $${w|}_{x=0}=0,{\frac{\partial w}{\partial x}|}_{x=0}=0$$

(4) $${w|}_{x=l}=0,{\frac{\partial^{2}w}{\partial x^{2}}|}_{x=l}=0$$

The assumed displacement solution satisfying the particular boundary conditions can be described in the form of [17] as Equation (5):

(5) $$w=\sum_{n=1}^{2}Y_{n}(t)X_{n}(x)$$

where $Y_{n}(t)$ denotes the amplitude of the mode, and the function $X_{n}(x)$ is determined by:

(6) $$\begin{array}{l} X_{n}=\cosh p_{n}x-\cos p_{n}x-\zeta_{n}(\sinh p_{n}x-\sin p_{n}x),\\ \zeta_{n}=\frac{\cosh p_{n}l+\cos p_{n}l}{\sinh p_{n}l+\sin p_{n}l},\\ p_{n}=\frac{(4n+1)\pi }{4l} \end{array}$$

Substituting Equation (2) with Equation (5), the vibration differential equations of the axially moving laminated SMA beam can be obtained by the dimensionless method, as follows:

(7) $$\begin{array}{ll} & -\rho b_{1}H\sum_{n=1}^{2}A_{ni}{\ddot{Y}}_{n}(t)+\sum_{n=1}^{2}\left(-2\rho b_{1}HvB_{ni}-cA_{ni}){\dot{Y}}_{n}(t\right)+\sum_{n=1}^{2}\{PC_{ni}-2\rho b_{1}Hv_{}^{2}C_{ni}-E\frac{b_{1}H^{3}}{12}D_{ni}\\ & -\frac{1}{2}b_{1}Hh[a(T-T_{M})(H+h)]\}Y_{n}(t)+\sum_{n=1}^{2}\frac{1}{2}Eb_{1}H^{2}E_{ni}Y_{n}^{2}+\frac{1}{2}Eb_{1}H^{2}S_{1i}Y_{1}Y_{2}+\sum_{n=1}^{2}[\frac{3}{2}Eb_{1}HF_{ni}-\frac{3}{8}b{\left(H+h\right)}^{3}(-b_{1}Hh)G_{ni}]Y_{n}^{3}\\ & +\frac{3}{2}Eb_{1}H(S_{2i}Y_{1}^{2}Y_{2}+S_{3i}Y_{1}Y_{2}^{2})-\frac{3}{8}b{\left(H+h\right)}^{3}(-b_{1}Hh)(S_{4i}Y_{1}^{2}Y_{2}+S_{5i}Y_{1}Y_{2}^{2})\\ & +\sum_{n=1}^{2}[\frac{5}{32}e{\left(H+h\right)}^{5}(-b_{1}Hh)H_{ni}]Y_{n}^{5}+\frac{5}{32}e{\left(H+h\right)}^{5}(-b_{1}Hh)(S_{6i}Y_{1}^{4}Y_{2}+S_{7i}Y_{1}Y_{2}^{4}+S_{8i}Y_{1}^{3}Y_{2}^{2}+S_{9i}Y_{1}^{2}Y_{2}^{3})=0 \end{array}$$

where the main coefficients are provided in Appendix A.

By introducing the non-dimensional parameters, $q_{n}=Y_{n}/L$, $H_{1}=h/H$, $H_{2}=H/L$, $E_{1}=\frac{a(T-T_{M})}{E}$, $E_{2}=\frac{b}{E}$, $E_{3}=\frac{e}{E}$, and simplifying the coefficients with the integral coefficients, the non-dimensional quantities of the laminated SMA beam are obtained:

(8) $$\begin{array}{l} {\ddot{q}}_{1}(t)+\omega_{1}^{2}q_{1}(t)+g_{1}^{2}q_{2}(t)+\mu_{11}{\dot{q}}_{1}(t)+\mu_{12}{\dot{q}}_{2}(t)+(a_{21}q_{1}^{2}+a_{22}q_{2}^{2}+a_{23}q_{1}q_{2})+(a_{31}q_{1}^{3}+a_{32}q_{2}^{3}\\ +a_{33}q_{1}^{2}q_{2}+a_{34}q_{1}q_{2}^{2})+(a_{51}q_{1}^{5}+a_{52}q_{2}^{5}+a_{53}q_{1}^{4}q_{2}+a_{54}q_{1}q_{2}^{4}+a_{55}q_{1}^{3}q_{2}^{2}+a_{56}q_{1}^{2}q_{2}^{3})=0 \end{array}$$

(9) $$\begin{array}{l} {\ddot{q}}_{2}(t)+\omega_{2}^{2}q_{2}(t)+g_{2}^{2}q_{1}(t)+\mu_{21}{\dot{q}}_{1}(t)+\mu_{22}{\dot{q}}_{2}(t)+(b_{21}q_{1}^{2}+b_{22}q_{2}^{2}+b_{23}q_{1}q_{2})+(b_{31}q_{1}^{3}+b_{32}q_{2}^{3}\\ +b_{33}q_{1}^{2}q_{2}+b_{34}q_{1}q_{2}^{2})+(b_{51}q_{1}^{5}+b_{52}q_{2}^{5}+b_{53}q_{1}^{4}q_{2}+b_{54}q_{1}q_{2}^{4}+b_{55}q_{1}^{3}q_{2}^{2}+b_{56}q_{1}^{2}q_{2}^{3})=0 \end{array}$$

where the coefficients are provided in Appendix B.

### 3.2. Application of Multi-Scale Method

In this part, the 1:3 internal resonance of the SMA laminated beam will be solved by the method of multiple scales [29].

After introducing a small parameter ε and different time variables, the approximate solution can be expressed as follows:

(10) $$\begin{array}{l} q_{1}=q_{11}(T_{0},T_{1})+\varepsilon q_{12}(T_{0},T_{1})\\ q_{2}=q_{21}(T_{0},T_{1})+\varepsilon q_{22}(T_{0},T_{1}) \end{array}$$

By substituting Equation (10) with Equations (8) and (9), after expansion, the coefficients of the same power term of ε were equal, and the approximate equation of $\varepsilon^{0}$ and $\varepsilon^{1}$ can be obtained. Approximate equations for $\varepsilon^{0}$:

(11) $$D_{0}^{2}q_{11}+\omega_{1}^{2}q_{11}=0$$

(12) $$D_{0}^{2}q_{21}+\omega_{2}^{2}q_{21}=0$$

Approximate equations for $\varepsilon^{1}$:

(13) $$\begin{array}{ll} D_{0}^{2}q_{12}+\omega_{1}^{2}q_{12}\;=\; & \left(-2D_{1}q_{11}-\mu_{11}q_{11}-\mu_{12}q_{21})D_{0}-(a_{21}q_{11}^{2}+a_{22}q_{21}^{2}+a_{23}q_{11}q_{21})-(a_{31}q_{11}^{3}+a_{32}q_{21}^{3}+a_{33}q_{11}^{2}q_{21}+a_{34}q_{11}q_{21}^{2}\right)\\ & -(a_{51}q_{11}^{5}+a_{52}q_{21}^{5}+a_{53}q_{11}^{4}q_{21}+a_{54}q_{11}q_{21}^{4}+a_{55}q_{11}^{3}q_{21}^{2}+a_{56}q_{11}^{2}q_{21}^{3}) \end{array}$$

(14) $$\begin{array}{ll} D_{0}^{2}q_{22}+\omega_{2}^{2}q_{22}\;=\; & \left(-2D_{1}q_{21}-\mu_{21}q_{11}-\mu_{22}q_{21})D_{0}-(b_{21}q_{11}^{2}+b_{22}q_{21}^{2}+b_{23}q_{11}q_{21})-(b_{31}q_{11}^{3}+b_{32}q_{21}^{3}+b_{33}q_{11}^{2}q_{21}+b_{34}q_{11}q_{21}^{2}\right)\\ & -(b_{51}q_{11}^{5}+b_{52}q_{21}^{5}+b_{53}q_{11}^{4}q_{21}+b_{54}q_{11}q_{21}^{4}+b_{55}q_{11}^{3}q_{21}^{2}+b_{56}q_{11}^{2}q_{21}^{3}) \end{array}$$

where, $D_{0}=\frac{\partial }{\partial T_{0}}$, $D_{1}=\frac{\partial }{\partial T_{1}}$, $D_{0}^{2}=\frac{\partial^{2}}{\partial T_{0}{}^{2}}$, $D_{1}^{2}=\frac{\partial^{2}}{\partial T_{1}{}^{2}}$.

The general solution of Equations (10) and (11) could be written as:

(15) $$q_{11}=A_{1}\left(T_{1}\right)e^{i\omega_{1}T_{0}}+{\bar{A}}_{1}\left(T_{1}\right)e^{-i\omega_{1}T_{0}}$$

(16) $$q_{21}=A_{2}\left(T_{1}\right)e^{i\omega_{2}T_{0}}+{\bar{A}}_{2}\left(T_{1}\right)e^{-i\omega_{2}T_{0}}$$

where $A_{1}\left(T_{1}\right)$ is a function at this point, ${\bar{A}}_{1}\left(T_{1}\right)$ is the conjugate of $A_{1}\left(T_{1}\right)$, and i is an imaginary unit. If we consider 1:3 internal resonance, after introducing the detuning parameter σ ($\omega_{2}=3\omega_{1}+\varepsilon \sigma$), we could obtain the following equation by substituting Equations (15) and (16) with Equations (13) and (14).

(17) $$\begin{array}{l} -2D_{1}A_{1}\omega_{1}i-\mu_{11}A_{1}\omega_{1}i-3a_{31}A_{1}^{2}{\bar{A}}_{1}-2a_{34}A_{1}A_{2}{\bar{A}}_{2}-10a_{54}A_{1}{}^{3}{\bar{A}}_{1}{}^{2}-6a_{54}A_{1}A_{2}{}^{2}{\bar{A}}_{2}{}^{2}\\ -6a_{55}A_{1}{}^{2}{\bar{A}}_{1}A_{2}{\bar{A}}_{2}+(a_{33}{\bar{A}}_{1}{}^{2}A_{2}-4a_{53}A_{1}{\bar{A}}_{1}{}^{3}A_{2}-3a_{56}{\bar{A}}_{1}{}^{2}A_{2}{}^{2}{\bar{A}}_{2})e^{i\sigma T_{1}}=0 \end{array}$$

(18) $$\begin{array}{l} -2D_{1}A_{2}\omega_{2}i-\mu_{22}A_{2}\omega_{2}i-3b_{32}A_{2}^{2}{\bar{A}}_{2}+2b_{33}A_{1}{\bar{A}}_{1}A_{2}-10b_{52}A_{2}{}^{3}{\bar{A}}_{2}{}^{2}-6b_{53}A_{1}{}^{2}{\bar{A}}_{1}{}^{2}A_{2}\\ -6b_{56}{\bar{A}}_{1}A_{2}{}^{2}{\bar{A}}_{2}+(-b_{31}A_{1}{}^{3}-5b_{51}A_{1}{}^{4}{\bar{A}}_{1}-2b_{55}{\bar{A}}_{1}{}^{3}A_{2}{\bar{A}}_{2})e^{i\sigma T_{1}}=0 \end{array}$$

In order to solve Equations (17) and (18), the complex function $A_{n}$ is written as:

(19) $$A_{n}\left(T_{1}\right)=\frac{1}{2}a_{n}\left(T_{1}\right)e^{i\beta_{n}}$$

where n=1,2, and $a_{n}$ and $\beta_{n}$ are real functions. 

Then, substituting Equation (19) with Equations (17) and (18), separating the real and imaginary parts, gave $\gamma =\beta_{2}-3\beta_{1}+\sigma T_{1}$, we obtain:

(20) $$2{a^{\prime }}_{1}\omega_{1}-\frac{1}{2}\mu_{11}a_{1}\omega_{1}+\frac{1}{8}a_{33}a_{1}^{2}a_{2}\sin\gamma -\frac{1}{8}a_{53}a_{1}^{4}a_{2}\sin\gamma -\frac{3}{32}a_{56}a_{1}^{2}a_{2}{}^{3}\sin\gamma =0$$

(21) $$2{a^{\prime }}_{2}\omega_{2}-\frac{1}{2}\mu_{22}a_{2}\omega_{2}-\frac{1}{8}b_{31}a_{1}^{3}\sin\gamma -\frac{5}{32}b_{51}a_{1}^{5}\sin\gamma -\frac{1}{16}b_{55}a_{1}^{3}a_{2}^{2}\sin\gamma =0$$

(22) $$2a_{1}{\beta^{\prime }}_{1}\omega_{1}-\frac{3}{8}a_{31}a_{1}^{3}+\frac{1}{8}a_{33}a_{1}{}^{2}a_{2}\cos\gamma -\frac{1}{4}a_{34}a_{1}a_{2}^{2}-\frac{5}{16}a_{51}a_{1}^{5}-\frac{1}{8}a_{53}a_{1}{}^{4}a_{2}\cos\gamma -\frac{3}{16}a_{55}a_{1}{}^{3}a_{2}{}^{2}-\frac{3}{32}a_{56}a_{1}{}^{2}a_{2}{}^{3}\cos\gamma =0$$

(23) $$2a_{2}{\beta^{\prime }}_{2}\omega_{2}-\;\frac{1}{8}b_{31}a_{1}^{3}\cos\gamma -\;\frac{3}{8}b_{32}a_{2}^{3}+\;\frac{1}{4}b_{33}a_{1}^{2}a_{2}-\frac{5}{32}b_{51}a_{1}^{5}\cos\gamma -\frac{5}{16}b_{52}a_{2}^{5}-\frac{3}{16}b_{53}a_{1}^{4}a_{2}-\frac{1}{16}b_{55}a_{1}^{3}a_{2}^{2}\cos\gamma -\frac{3}{16}b_{56}a_{1}^{2}a_{2}^{3}=0$$

Then $\beta_{1}$ and $\beta_{2}$ can be eliminated by the Equations (22) and (23), we get:

(24) $$\begin{array}{ll} a_{2}\gamma^{\prime } & =a_{2}\sigma +\left(\frac{3a_{34}}{8\omega_{1}}-\frac{3b_{32}}{16\omega_{2}}\right)a_{2}^{3}+\left(\frac{9a_{31}}{16\omega_{1}}+\;\frac{b_{33}}{8\omega_{2}}\right)a_{1}^{2}a_{2}+\;\left(\frac{9a_{54}}{32\omega_{1}}-\frac{5b_{52}}{32\omega_{2}}\right)a_{2}^{5}+\left(\frac{15a_{51}}{32\omega_{1}}-\frac{3b_{53}}{32\omega_{2}}\right)a_{1}^{4}a_{2}+\left(\frac{9a_{55}}{32\omega_{1}}-\frac{3b_{56}}{32\omega_{2}}\right)a_{1}^{2}a_{2}^{3}\\ & +\left(-\frac{3a_{33}a_{1}a_{2}^{2}}{16\omega_{1}}-\frac{b_{31}a_{1}^{3}}{16\omega_{2}}\right)\cos\gamma +\left(\frac{3a_{53}}{16\omega_{1}}-\frac{b_{55}}{32\omega_{2}}\right)a_{1}^{3}a_{2}^{2}\cos\gamma +\left(\frac{9a_{56}a_{1}a_{2}^{4}}{64\omega_{1}}-\frac{5b_{51}a_{1}^{5}}{64\omega_{2}}\right)\cos\gamma \end{array}$$

To analyze the steady-state motion of the 1:3 internal resonance, we can obtain the equation ${a^{\prime }}_{1}={a^{\prime }}_{2}=\gamma^{\prime }=0$ from Equations (20)–(24).

## 4. Influence of Parameters for the Internal Resonances of Laminated SMA Beam

The nonlinear dynamic analytical model is cast into state-space form, non-dimensionalized, and then solved numerically into the time domain using a fourth-order Runge–Kutta scheme. The MATLAB programming is used to solve the dimensionless amplitude modalities—$a_{1}$ and $a_{2}$, and carried on computer simulation.

For the axially moving laminated SMA beam, let L=0.5 m, $b_{1}=0.05\;m$, H=0.02 m, E=206 GPa, $\rho =7900\;\mathrm{kg}/m^{3}$, and T=300 K. In Table 1, the first and second natural frequencies of the system corresponding to different axial velocities and the thickness ratio of the SMA layer to the base beam ($H_{1}=\frac{h}{H}$) are given. It can be found that the first two natural frequencies decrease slightly with the increase in axial velocity, and the first two natural frequencies also decrease slightly with the decrease in SMA layer thickness.

### 4.1. System without Damping

**Figure 3:**
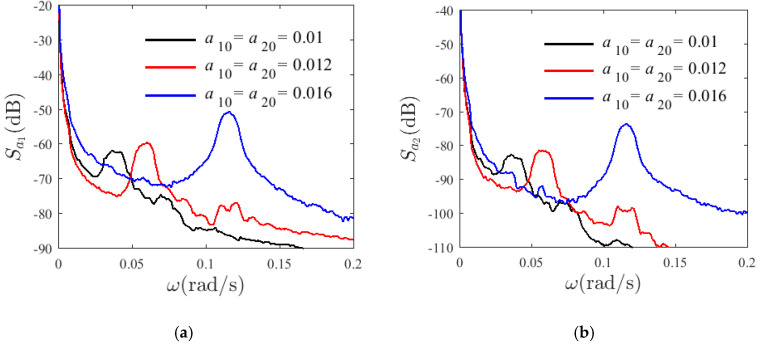
PSD curves of variation modes for different initial conditions a ($\gamma_{0}=0.2\pi$); (**a**) $a_{1}$, (**b**) $a_{2}$.

Resonance is very sensitive to the initial conditions. For v=0 m/s, $H_{1}=0.22$, and T=300 K, Figure 2 and Figure 3 show the time-history and the spectrum of a free, un-damping nonlinear vibration system with different initial conditions and dimensionless amplitude modalities—$a_{1}$ and $a_{2}$. Figure 2 shows the modal amplitude chart with different initial values: $a_{10}=a_{20}=0.01$ (black), $a_{10}=a_{20}=0.012$ (red), and $a_{10}=a_{20}=0.016$ (blue). The 1:3 internal resonance can be observed in the system and its energy varies from the first-order mode (solid line) to second-mode (dashed line), presenting a periodic motion. Moreover, the fluctuation range of the first-order modal amplitude is greater than that of the second-order modal. As can be seen from the spectrogram, with the increase in the initial value, the vibration frequency of a second mode of the system also increases, illustrating the nonlinear vibration characteristics of the system.

**Figure 4:**
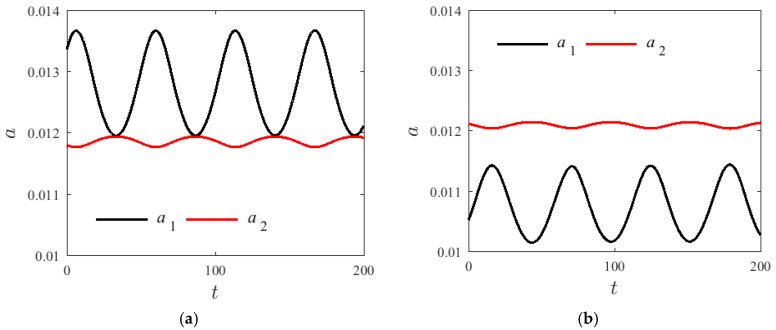
Variation modes for different initial conditions γ ($a_{10}=a_{20}=0.012$); (**a**) $\gamma_{0}=0$, (**b**) $\gamma_{0}=2\pi$.

Figure 4 shows a graph of the changes of amplitude over time for two different groups $a_{1}$ and $a_{2}$ with v=0 m/s, $H_{1}=0.22$, and T=300 K. The initial value of the modal amplitude is $a_{10}=a_{20}=0.012$. The initial value increases from 0 to 2π, the fluctuation region of the first-order modal amplitude reduced with the increase in $\gamma_{0}$, but the vibration frequency of the first and second modes do not change significantly. A similar conclusion was also found in [17].

**Figure 5:**
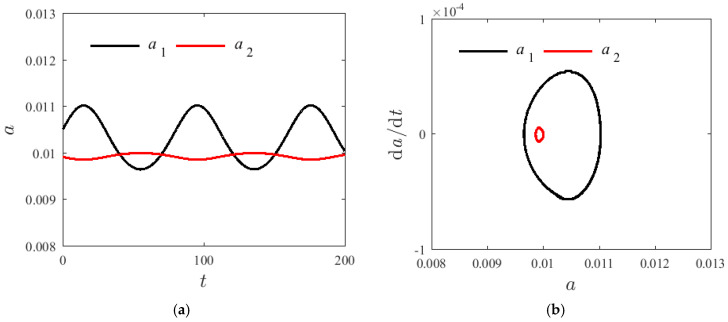
Internal resonances response curves v=0; (**a**) time process diagram, (**b**) phase diagrams.

**Figure 6:**
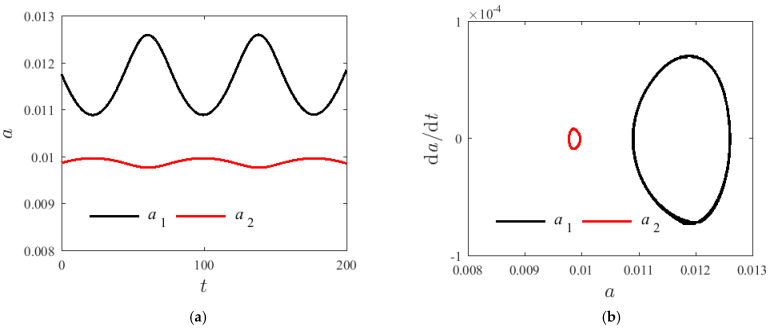
Internal resonances response curves v=50 m/s; (**a**) time process diagram, (**b**) phase diagrams.

**Figure 7:**
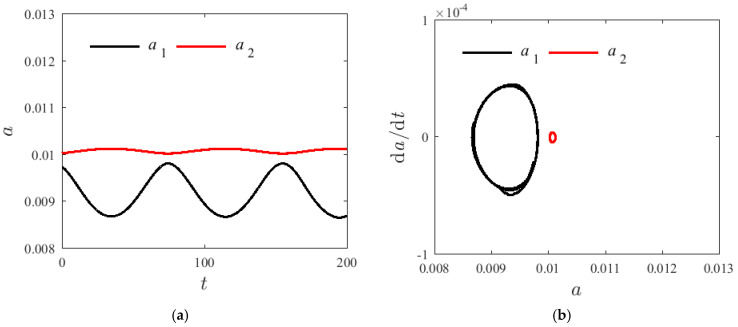
Internal resonances response curves v=100 m/s; (**a**) time process diagram, (**b**) phase diagrams.

Figure 5, Figure 6 and Figure 7 demonstrate the time spectrum when $H_{1}=0.22$, T=300 K, and σ=0.001. The initial value of the amplitude are respectively $a_{10}=a_{20}=0.01$ and $\gamma_{0}=0.5\pi$ with changing axial speeds v=0, v=50 m/s, and v=100 m/s. With the increase in the axial velocity, the fluctuation range of a post-order mode amplitude increases firstly and then decreases; however, the fluctuation range of the amplitude of the second-order mode shows no significant change. Figure 6 and Figure 7 illustrate that the second mode can suddenly overtake the first mode modal amplitude with a greater axial velocity. The enclosed ring in the phase diagram represents the second mode; the left side of the ring changes to the right side, and the axial velocity has an effect on the resonance characteristics of the system. However, when the axial velocity changes, the vibration frequency of the first-order mode shows no significant change.

### 4.2. Damped System

**Figure 8:**
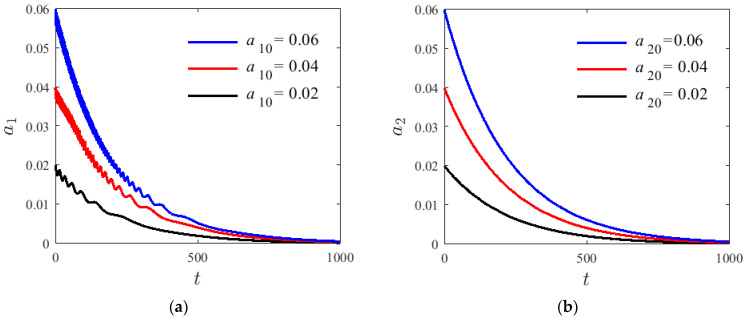
The decay graphs of variation modes for different initial conditions; (**a**) modal amplitude of $a_{1}$, (**b**) modal amplitude of $a_{1}$.

**Figure 9:**
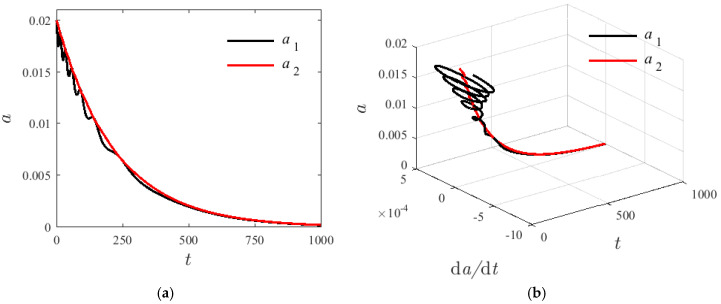
The coupling decay graphs of variation modes; (**a**) time-history response diagrams, (**b**) 3D response diagram.

For the damping system, numerical methods are used to analyze characteristic vibration variations of the first two step changes when resonance occurs, and to discuss the parameter influence, given $H_{1}=0.22$, T=300 K, σ=0.01, v=80 m/s, and $\gamma_{0}=0.1\pi$. Figure 8 shows the modal amplitude of $a_{1}$ and $a_{2}$ in a damped free-vibration system with internal resonance at different initial values. The decay rate of the mode amplitude is increased with the increase in the initial value of $a_{1}$ and $a_{2}$. As modal amplitude fluctuation frequency increases, the second mode of amplitude fluctuation is not obvious leading to a zero attenuation. The damping term is determined by the axial velocity and the damping coefficient of the base beam. Figure 9 shows that in a damped internal resonance system, the first- and second-order vibration modes exhibit a tendency of coupling attenuation until zero and eventually increase with time.

## 5. Conclusions

This study focused on the internal resonance of the 1:3 SMA laminated beam moving in complex boundary conditions. The lateral vibration equations were derived with a multiple scales method. Through numerical simulations, the resonance problems interposed with the SMA laminated beam clamped at one end and hinge-joined at the other end were analyzed. The results showed: 

(1) For un-damping the nonlinear system, the system has periodic motion. The system energy is exchanged between the two coupled vibration modes, but this periodic behavior is not stable, showing an obvious dependence of the initial value. 

(2) In the steady state solution of the un-damping nonlinear system, along with the increase in the axial velocity, the fluctuation range of the first-mode amplitude increases and then decreases. However, the fluctuation range of the amplitude of the second-order mode shows no significant change. 

(3) In a damped system, the vibration mode of the system shows a tendency to decrease to attenuation. Moreover, the attenuation rate of the modal amplitude increases with the increase in the initial value of $a_{1}$ and $a_{2}$.

## Figures and Tables

**Table 1:** Natural frequency and thickness ration of SMA to base beam.

Axial Velocity m/s	h/H	$w_{1}$	$w_{2}$
v=0 m/s	0.35	0.5546	1.6630
v=40 m/s	0.32	0.5503	1.6502
v=80 m/s	0.22	0.5372	1.6112

## Appendix A

Main coefficients in Equation (7).

$$\begin{array}{ll} A_{ni}= & \int_{0}^{l}X_{n}X_{i}dx,B_{ni}=\int_{0}^{l}\frac{dX_{n}}{dx}X_{i}dx,C_{ni}=\int_{0}^{l}\frac{d^{2}X_{n}}{dx^{2}}X_{i}dx,D_{ni}=\int_{0}^{l}\frac{d^{4}X_{n}}{dx^{4}}X_{i}dx,\\ E_{ni}= & \int_{0}^{l}[\frac{dX_{n}}{dx}\frac{d^{3}X_{n}}{dx^{3}}+{\left(\frac{d^{2}X_{n}}{dx^{2}}\right)}^{2}]X_{i}dx,\\ F_{ni}= & \int_{0}^{l}{\left(\frac{dX_{n}}{dx}\right)}^{2}\frac{d^{2}X_{n}}{dx^{2}}X_{i}dx,\\ G_{ni}= & \int_{0}^{l}[{\left(\frac{dX_{n}}{dx}\right)}^{2}\frac{d^{4}X_{n}}{dx^{4}}+\frac{d^{2}X_{n}}{dx^{2}}{\left(\frac{d^{3}X_{n}}{dx^{3}}\right)}^{2}]X_{i}dx,\\ H_{ni}= & \int_{0}^{l}[{\left(\frac{d^{2}X_{n}}{dx^{2}}\right)}^{4}\frac{d^{4}X_{n}}{dx^{4}}+{\left(\frac{d^{2}X_{n}}{dx^{2}}\right)}^{3}{\left(\frac{d^{3}X_{n}}{dx^{3}}\right)}^{2}]X_{i}dx,\\ S_{1i}= & \int_{0}^{l}[\frac{dX_{1}}{dx}\frac{d^{3}X_{2}}{dx^{3}}+\frac{dX_{2}}{dx}\frac{d^{3}X_{1}}{dx^{3}}]X_{i}dx,\\ S_{2i}= & \int_{0}^{l}[{\left(\frac{d^{2}X_{1}}{dx^{2}}\right)}^{2}\frac{dX_{2}}{dx}+\frac{1}{2}\frac{dX_{1}}{dx}\frac{dX_{2}}{dx}\frac{d^{2}X_{1}}{dx^{2}}]X_{i}dx,\\ S_{3i}= & \int_{0}^{l}[{\left(\frac{d^{2}X_{2}}{dx^{2}}\right)}^{2}\frac{dX_{1}}{dx}+\frac{1}{2}\frac{dX_{1}}{dx}\frac{dX_{2}}{dx}\frac{d^{2}X_{2}}{dx^{2}}]X_{i}dx,\\ S_{4i}= & \int_{0}^{l}[{\left(\frac{d^{2}X_{1}}{dx^{2}}\right)}^{2}\frac{d^{4}X_{2}}{dx^{4}}+\frac{1}{2}\frac{d^{2}X_{2}}{dx^{2}}{\left(\frac{d^{3}X_{1}}{dx^{43}}\right)}^{2}+\frac{1}{2}\frac{d^{2}X_{1}}{dx^{2}}\frac{d^{2}X_{2}}{dx^{2}}\frac{d^{4}X_{1}}{dx^{4}}+\frac{1}{4}\frac{d^{2}X_{1}}{dx^{2}}\frac{d^{3}X_{1}}{dx^{3}}\frac{d^{3}X_{3}}{dx^{3}}]X_{i}dx,\\ S_{5i}= & \int_{0}^{l}[{\left(\frac{d^{2}X_{2}}{dx^{2}}\right)}^{2}\frac{d^{4}X_{1}}{dx^{4}}+\frac{1}{2}\frac{d^{2}X_{1}}{dx^{2}}{\left(\frac{d^{3}X_{2}}{dx^{3}}\right)}^{2}+\frac{1}{2}\frac{d^{2}X_{1}}{dx^{2}}\frac{d^{2}X_{2}}{dx^{2}}\frac{d^{4}X_{2}}{dx^{4}}+\frac{1}{4}\frac{d^{2}X_{2}}{dx^{2}}\frac{d^{3}X_{1}}{dx^{3}}\frac{d^{3}X_{2}}{dx^{3}}]X_{i}dx,\\ S_{6i}= & \int_{0}^{l}[{\left(\frac{d^{4}X_{1}}{dx^{4}}\right)}^{4}\frac{d^{4}X_{2}}{dx^{4}}+\frac{1}{4}{\left(\frac{d^{2}X_{2}}{dx^{2}}\right)}^{3}\frac{d^{2}X_{2}}{dx^{2}}\frac{d^{4}X_{1}}{dx^{4}}+\frac{1}{8}{\left(\frac{d^{2}X_{1}}{dx^{2}}\right)}^{3}\frac{d^{3}X_{1}}{dx^{3}}\frac{d^{3}X_{2}}{dx^{3}}\\ & +\frac{1}{12}{\left(\frac{d^{2}X_{1}}{dx^{2}}\right)}^{2}\frac{d^{2}X_{2}}{dx^{2}}{\left(\frac{d^{3}X_{1}}{dx^{3}}\right)}^{2}]X_{i}dx,\\ S_{7i}= & \int_{0}^{l}[{\left(\frac{d^{2}X_{2}}{dx^{2}}\right)}^{4}\frac{d^{4}X_{1}}{dx^{4}}+\frac{1}{4}\frac{d^{2}X_{1}}{dx^{2}}{\left(\frac{d^{2}X_{2}}{dx^{2}}\right)}^{3}\frac{d^{4}X_{2}}{dx^{4}}+\frac{1}{8}{\left(\frac{d^{2}X_{2}}{dx^{2}}\right)}^{3}\frac{d^{3}X_{1}}{dx^{3}}\frac{d^{3}X_{2}}{dx^{3}}\\ & +\frac{1}{12}\frac{d^{2}X_{1}}{dx^{2}}{\left(\frac{d^{2}X_{2}}{dx^{2}}\right)}^{2}{\left(\frac{d^{3}X_{2}}{dx^{3}}\right)}^{2}]X_{i}dx,\\ S_{8i}= & \int_{0}^{l}[\frac{1}{4}{\left(\frac{d^{2}X_{1}}{dx^{2}}\right)}^{3}\frac{d^{2}X_{2}}{dx^{2}}\frac{d^{4}X_{2}}{dx^{4}}+\frac{1}{4}{\left(\frac{d^{2}X_{1}}{dx^{2}}\right)}^{3}{\left(\frac{d^{3}X_{2}}{dx^{3}}\right)}^{2}+\frac{1}{6}{\left(\frac{d^{2}X_{1}}{dx^{2}}\right)}^{2}{\left(\frac{d^{2}X_{2}}{dx^{2}}\right)}^{2}\frac{d^{4}X_{1}}{dx^{4}}\\ & +\frac{1}{12}\frac{d^{2}X_{1}}{dx^{2}}{\left(\frac{d^{2}X_{2}}{dx^{2}}\right)}^{2}{\left(\frac{d^{3}X_{2}}{dx^{3}}\right)}^{2}+\frac{1}{24}{\left(\frac{d^{2}X_{1}}{dx^{2}}\right)}^{2}\frac{d^{2}X_{2}}{dx^{2}}\frac{d^{3}X_{1}}{dx^{3}}\frac{d^{3}X_{2}}{dx^{3}}]X_{i}dx,\\ S_{9i}= & \int_{0}^{l}[\frac{1}{4}{\left(\frac{d^{2}X_{1}}{dx^{2}}\right)}^{3}\frac{d^{2}X_{2}}{dx^{2}}\frac{d^{4}X_{1}}{dx^{4}}+\frac{1}{4}{\left(\frac{d^{2}X_{2}}{dx^{2}}\right)}^{3}{\left(\frac{d^{3}X_{1}}{dx^{3}}\right)}^{2}+\frac{1}{6}{\left(\frac{d^{2}X_{1}}{dx^{2}}\right)}^{2}{\left(\frac{d^{2}X_{2}}{dx^{2}}\right)}^{2}\frac{d^{4}X_{2}}{dx^{4}}\\ & +\frac{1}{12}\frac{d^{2}X_{2}}{dx^{2}}{\left(\frac{d^{2}X_{1}}{dx^{2}}\right)}^{2}{\left(\frac{d^{3}X_{2}}{dx^{3}}\right)}^{2}+\frac{1}{24}{\left(\frac{d^{2}X_{2}}{dx^{2}}\right)}^{2}\frac{d^{2}X_{1}}{dx^{2}}\frac{d^{3}X_{1}}{dx^{3}}\frac{d^{3}X_{2}}{dx^{3}}]X_{i}dx.\\ \omega_{1}^{2}= & [\frac{E_{1}EH_{1}H_{2}^{2}L^{2}(1+H_{1})}{\rho }+\frac{1}{12}\frac{EH_{2}^{2}L^{2}}{\rho }]\frac{D_{11}}{A_{11}}+(2v-\frac{P}{\rho bH_{2}L})\frac{C_{11}}{A_{11}},\\ g_{1}^{2}= & [\frac{E_{1}EH_{1}H_{2}^{2}L^{2}(1+H_{1})}{\rho }+\frac{1}{12}\frac{EH_{2}^{2}L^{2}}{\rho }]\frac{D_{21}}{A_{11}}+(2v-\frac{P}{\rho bH_{2}L})\frac{C_{21}}{A_{11}},\\ g_{2}^{2}= & [\frac{E_{1}EH_{1}H_{2}^{2}L^{2}(1+H_{1})}{\rho }+\frac{1}{12}\frac{EH_{2}^{2}L^{2}}{\rho }]\frac{D_{12}}{A_{22}}+(2v-\frac{P}{\rho bH_{2}L})\frac{C_{12}}{A_{22}}. \end{array}$$

## Appendix B

Main coefficients in Equations (8) and (9).

$$\begin{array}{ll} \mu_{11}= & 2v\frac{B_{11}}{A_{11}}+\frac{c}{\rho b_{1}LH_{2}},\mu_{12}=2v\frac{B_{21}}{A_{11}}+\frac{cA_{21}}{\rho b_{1}LH_{2}A_{11}},\\ \mu_{21}= & 2v\frac{B_{12}}{A_{22}}+\frac{cA_{12}}{\rho b_{1}LH_{2}A_{22}},\mu_{22}=2v\frac{B_{22}}{A_{22}}+\frac{c}{\rho b_{1}LH_{2}},\\ a_{21}= & -\frac{1}{2}\frac{EH_{2}L^{2}E_{11}}{\rho A_{11}},a_{22}=-\frac{1}{2}\frac{EH_{2}L^{2}E_{21}}{\rho A_{11}},a_{23}=-\frac{1}{2}\frac{EH_{2}L^{2}S_{11}}{\rho A_{11}},\\ b_{21}= & -\frac{1}{2}\frac{EH_{2}L^{2}E_{12}}{\rho A_{22}},b_{22}=-\frac{1}{2}\frac{EH_{2}L^{2}E_{22}}{\rho A_{22}},b_{23}=-\frac{1}{2}\frac{EH_{2}L^{2}S_{12}}{\rho A_{22}},\\ a_{31}= & -\frac{3}{2}\frac{EL^{2}F_{11}}{\rho A_{11}}-\frac{3}{8}\frac{E_{2}EH_{1}H_{2}^{4}L^{6}{\left(1+H_{1}\right)}^{3}G_{11}}{\rho A_{11}},\\ a_{32}= & -\frac{3}{2}\frac{EL^{2}F_{21}}{\rho A_{11}}-\frac{3}{8}\frac{E_{2}EH_{1}H_{2}^{4}L^{6}{\left(1+H_{1}\right)}^{3}G_{21}}{\rho A_{11}},\\ a_{33}= & -\frac{3}{2}\frac{EL^{2}S_{21}}{\rho A_{11}}-\frac{3}{8}\frac{E_{2}EH_{1}H_{2}^{4}L^{6}{\left(1+H_{1}\right)}^{3}S_{41}}{\rho A_{11}},\\ a_{34}= & -\frac{3}{2}\frac{EL^{2}S_{31}}{\rho A_{11}}-\frac{3}{8}\frac{E_{2}EH_{1}H_{2}^{4}L^{6}{\left(1+H_{1}\right)}^{3}S_{51}}{\rho A_{11}},\\ b_{31}= & -\frac{3}{2}\frac{EL^{2}F_{12}}{\rho A_{22}}-\frac{3}{8}\frac{E_{2}EH_{1}H_{2}^{4}L^{6}{\left(1+H_{1}\right)}^{3}G_{12}}{\rho A_{22}},\\ b_{32}= & -\frac{3}{2}\frac{EL^{2}F_{22}}{\rho A_{22}}-\frac{3}{8}\frac{E_{2}EH_{1}H_{2}^{4}L^{6}{\left(1+H_{1}\right)}^{3}G_{22}}{\rho A_{22}},\\ b_{33}= & -\frac{3}{2}\frac{EL^{2}S_{22}}{\rho A_{22}}-\frac{3}{8}\frac{E_{2}EH_{1}H_{2}^{4}L^{6}{\left(1+H_{1}\right)}^{3}S_{42}}{\rho A_{22}},\\ b_{34}= & -\frac{3}{2}\frac{EL^{2}S_{32}}{\rho A_{22}}-\frac{3}{8}\frac{E_{2}EH_{1}H_{2}^{4}L^{6}{\left(1+H_{1}\right)}^{3}S_{52}}{\rho A_{22}},\\ a_{51}= & \frac{5}{32}\frac{E_{3}EH_{1}H_{2}^{6}L^{10}{\left(1+H_{1}\right)}^{5}H_{11}}{\rho A_{11}},a_{52}=\frac{5}{32}\frac{E_{3}EH_{1}H_{2}^{6}L^{10}{\left(1+H_{1}\right)}^{5}H_{21}}{\rho A_{11}},\\ a_{53}= & \frac{5}{32}\frac{E_{3}EH_{1}H_{2}^{6}L^{10}{\left(1+H_{1}\right)}^{5}S_{61}}{\rho A_{11}},a_{54}=\frac{5}{32}\frac{E_{3}EH_{1}H_{2}^{6}L^{10}{\left(1+H_{1}\right)}^{5}S_{71}}{\rho A_{11}},\\ a_{55}= & \frac{5}{32}\frac{E_{3}EH_{1}H_{2}^{6}L^{10}{\left(1+H_{1}\right)}^{5}S_{81}}{\rho A_{11}},a_{56}=\frac{5}{32}\frac{E_{3}EH_{1}H_{2}^{6}L^{10}{\left(1+H_{1}\right)}^{5}S_{91}}{\rho A_{11}},\\ b_{51}= & \frac{5}{32}\frac{E_{3}EH_{1}H_{2}^{6}L^{10}{\left(1+H_{1}\right)}^{5}H_{12}}{\rho A_{22}},b_{52}=\frac{5}{32}\frac{E_{3}EH_{1}H_{2}^{6}L^{10}{\left(1+H_{1}\right)}^{5}H_{22}}{\rho A_{22}},\\ b_{53}= & \frac{5}{32}\frac{E_{3}EH_{1}H_{2}^{6}L^{10}{\left(1+H_{1}\right)}^{5}S_{62}}{\rho A_{22}},b_{54}=\frac{5}{32}\frac{E_{3}EH_{1}H_{2}^{6}L^{10}{\left(1+H_{1}\right)}^{5}S_{72}}{\rho A_{22}},\\ b_{55}= & \frac{5}{32}\frac{E_{3}EH_{1}H_{2}^{6}L^{10}{\left(1+H_{1}\right)}^{5}S_{82}}{\rho A_{22}},b_{56}=\frac{5}{32}\frac{E_{3}EH_{1}H_{2}^{6}L^{10}{\left(1+H_{1}\right)}^{5}S_{92}}{\rho A_{22}}. \end{array}$$

## References

[1] Mirzaeifar R., Desroches R., Yavari A. (2011). A combined analytical, numerical, and experimental study of shape-memory-alloy helical springs. Int. J. Solids Struct..

[2] Pacheco P.M.C.L., Machado L.G., Savi M.A. (2003). Nonlinear dynamics and chaos in coupled shape memory oscillators. Int. J. Solids Struct..

[3] Savi M.A., Pacheco P.M.C.L., Braga A.M.B. (2002). Chaos in a shape memory two-bar truss. Int. J. Non Linear Mech..

[4] Ren Y., Shao B. (2003). Analysis of free vibrations of shape memory alloy hybrid composite beams. Eng. Mech..

[5] Shao B., Ren Y.S. (2004). The semi-active control of shape memory alloy composite beam. Mech. Eng..

[6] Ren Y.S., Liu Y.R., Yang S., Wang X. (2010). Active deformation models of SMA fiber hybrid thin-walled laminated beams. Chin. J. Solid Mech..

[7] Collet M., Foltête E., Lexcellent C. (2001). Analysis of the behavior of a Shape Memory Alloy beam under dynamical loading. Eur. J. Mech. Solids.

[8] de Matos Junior O.D., Donadon M.V., Castro S.G.P. (2017). Aeroelastic behavior of stiffened composite laminated panel with embedded SMA wire using the hierarchical Rayleigh–Ritz method. Compos. Struct..

[9] Akhavan-Rad B., Kheirikhah M.M. (2019). Static analysis of sandwich plates embedded with shape memory alloy wires using active strain energy tuning method. J. Braz. Soc. Mech. Sci. Eng..

[10] Razavilar R., Fathi A., Dardel M., Hadi J.A. (2018). Dynamic analysis of a shape memory alloy beam with pseudoelastic behavior. J. Intell. Mater. Syst. Struct..

[11] Lu P., Cui F.S., Tan M.J. (2009). A theoretical model for the bending of a laminated beam with SMA fiber embedded layer. Compos. Struct..

[12] Zhang Z. (2012). Dynamic Bifurcation and Control of the Structures with Shape Memory Alloy (SMA). Ph.D. Thesis.

[13] Fu S., Lu Q. (2012). Nonlinear dynamics and vibration reduction of a dry friction oscillator with SMA restraints. Nonlinear Dyn..

[14] Asadi H., Bodaghi M., Shakeri M., Aghdam M. (2013). On the free vibration of thermally pre/post-buckled shear deformable SMA hybrid composite beams. Aerosp. Sci. Technol..

[15] Samadpour M., Asadi H., Wang Q. (2016). Nonlinear aero-thermal flutter postponement of supersonic laminated composite beams with shape memory alloys. Eur. J. Mech. Solids.

[16] Zhang X., Gao M., Hao Y. (2021). The 1/3rd subharmonic and 3rd superharmonic resonance of a shape memory alloy (SMA) laminated beam. J. Theor. Appl. Mech..

[17] Wang J., Hu Y., Su Y., Gong L. (2019). Magneto-elastic internal resonance of an axially moving conductive beam in the magnetic field. J. Theor. Appl. Mech..

[18] Zhang K., Tan X., Ding H., Chen L. (2018). Parametric Vibration Responses of Supercritical Fluid-Conveying Pipes in 3:1 Internal Resonance. Appl. Math. Mech..

[19] Yang J., Zhang W., Xi A. (2018). Nonlinear Dynamics Analysis of a Composite Cantilever Piezoelectric Plate with One-To-Three Internal Resonance. J. Dyn. Control.

[20] Zhang D.B., Tang Y.Q., Liang R.Q., Yang L., Chen L.Q. (2021). Dynamic stability of an axially transporting beam with two-frequency parametric excitation and internal resonance. Eur. J. Mech. Solids.

[21] Wang J., Zhu Y., Zhang B., Shen H., Liu J. (2020). Nonlocal and strain gradient effects on nonlinear forced vibration of axially moving nanobeams under internal resonance conditions. Appl. Math. Mech..

[22] Zhang D.-B., Tang Y.-Q., Chen L.-Q. (2019). Internal resonance in parametric vibrations of axially accelerating viscoelastic plates. Eur. J. Mech. Solids.

[23] Mao X.-Y., Ding H., Chen L.-Q. (2019). Internal resonance of a supercritically axially moving beam subjected to the pulsating speed. Nonlinear Dyn..

[24] Ding H., Huang L., Chen L. (2017). Primary resonance of traveling viscoelastic beam under internal resonance. Appl. Math. Mech..

[25] Zhu B., Dong Y., Li Y. (2018). Nonlinear dynamics of a viscoelastic sandwich beam with parametric excitations and internal resonance. Nonlinear Dyn..

[26] Sahoo B., Panda L.N., Pohit G. (2017). Stability, Bifurcation and Chaos of a Traveling Viscoelastic Beam Tuned to 3:1 Internal Resonance and Subjected to Parametric Excitation. Int. J. Bifurc. Chaos.

[27] Paiva A., Savi M.A. (2006). An overview of constitutive models for shape memory alloys. Math. Probl. Eng..

[28] Ying H., Minglei G. (2019). Traverse Vibration of Axially Moving Laminated SMA Beam considering Random Perturbation. Shock Vib..

[29] Hu Y., Wang J. (2017). Principal-internal resonance of an axially moving current-carrying beam in magnetic field. Nonlinear Dyn..

